# Editorial: Affective Learning in Digital Education

**DOI:** 10.3389/fpsyg.2020.630966

**Published:** 2021-01-28

**Authors:** Andreas Gegenfurtner, Susanne Narciss, Luke K. Fryer, Sanna Järvelä, Judith M. Harackiewicz

**Affiliations:** ^1^Department of Educational Science, University of Regensburg, Regensburg, Germany; ^2^Psychology of Learning and Instruction, Faculty of Psychology, Technische Universität Dresden, Dresden, Germany; ^3^Faculty of Education, Centre of the Enhancement of Teaching and Learning, The University of Hong Kong, Pokfulam, Hong Kong; ^4^Department of Educational Sciences and Teacher Education, University of Oulu, Oulu, Finland; ^5^Department of Psychology, University of Wisconsin-Madison, Madison, WI, United States

**Keywords:** digital education, motivation, emotion, affect, learning, technology

Digital education opens up novel avenues for students and teachers to learn and interact together. The evolution of digital tools for learning and the constant transformation of educational technology inspire research that seeks to understand how students' adaptive motivations and emotions for learning might be supported and how learning environments can be personalized. There is an urgent need for evidence-based development, as the recent COVID19 pandemic has led to the use of online and digital tools in education at all levels. These tools include webinars (Ebner and Gegenfurtner), digital games (Näykki et al.), hypermedia-based tutoring systems (Wortha et al.), virtual laboratories (Pietarinen et al.), adaptive learning environments (Molenaar et al.), social networks (Näykki et al.), and online courses (Francis et al.; Knigge et al.; Quesada-Pallarès et al.; Stephan et al.). These are embedded within asynchronous, blended, hybrid, interactive, mobile, online, synchronous, virtual, or web-based learning environments. When we seek to understand how and why students learn in these digital education scenarios, then a focus on students' affective processes is particularly useful, where “affective” is understood as an umbrella term to include processes such as the motivations, intentions, emotions, interests, satisfaction, values, goals, and attitudes of leaners, which can be individually or socially regulated.

This Research Topic brings together studies on the nexus of motivation science and educational technology to explore affective learning in digitally mediated scenarios. Aiming to expand what we know about learning and motivation in digital contexts, this Research Topic has three main objectives. The first is to deepen our understanding of how learning and motivation processes interrelate and co-evolve in digital environments. For example, situated in a hypermedia-based tutoring system, Wortha et al. find that positive emotion pattern scores before the learning activity and negative emotion pattern scores during the learning activity predicted learning, but not consistently. Similarly, Testers et al., in a study on non-traditional students in asynchronous online education, reported that the motivation to learn, expected positive personal outcomes, and learner readiness were the strongest predictors of transfer intentions. These and other studies in this Research Topic explore the interrelations between motivation and learning in digital education.

A second objective of the Research Topic is to evaluate how effective digital tools, media, and infrastructure are in supporting affective learning. The study by Molenaar et al. demonstrates that young learners need performance feedback to support correct self-evaluation and drive their regulatory actions in adaptive learning environments. Knigge et al. note a significant increase in empathic concern after working with video-based online teacher training. Focusing on the differences between digital and face-to-face learning environments, Stephan et al. show that teacher education students attending an asynchronous online course (compared to a synchronous face-to-face course) reported a higher level of boredom, anxiety, and anger, but less enjoyment. This finding is echoed by Ebner and Gegenfurtner who indicate meta-analytically that learners in asynchronous online courses are less satisfied than in synchronous webinars. However, Francis et al. and Quesada-Pallarès et al., report that online and face-to-face students may differ overall in academic outcomes but not in their motivation, task value, or metacognitive self-regulation. These studies are fascinating, as they address individual differences and the effectiveness of digital tools to support affective learning.

Finally, a third objective of the Research Topic is to review and evaluate the development of frontline innovations in the methods, measures, and technologies used for the investigation and promotion of the processes and products of affective learning. It presents a number of emerging multimodal methods that use digital tools for data collection and analysis. These tools and measures include, but are not limited to, wearables, handhelds, heart rate measures, as well as electroencephalographic and electrodermal activity (Järvenoja et al.; Martens et al.; Näykki et al.). We also see detailed interaction analyses of collaborative learning processes and the socially shared regulation of learning and emotion (Näykki et al.; Pietarinen et al.). Furthermore, Wortha et al. describe an innovative integration of person-centered and variable-centered approaches to cluster analysis. Overall, the studies in this Research Topic illustrate multimodal and multimethod analyses of affective multichannel data in digital learning contexts.

These three objectives are relevant for research that addresses digital education for people of different ages—from learners in early childhood education (Näykki et al.) to K12 (Järvenoja et al.; Molenaar et al.; Näykki et al.; Pietarinen et al.) and higher education (Ebner and Gegenfurtner; Francis et al.; Knigge et al.; Martens et al.; Näykki et al.; Stephan et al.; Testers et al.; Wortha et al.) up to vocational and professional training contexts (Ebner and Gegenfurtner; Quesada-Pallarès et al.)—and for studies that are situated within various disciplinary fields, such as science education (Francis et al.; Pietarinen et al.), maker education (Näykki et al.), medical education (Ebner and Gegenfurtner), and teacher education (Knigge et al.; Stephan et al.). A total of twelve studies addressed these objectives using empirical data from learners studying in a number of countries, including Finland, Germany, Spain, the Netherlands, and the US. Table 1 provides an overview of studies contributing to this Research Topic.

**Table 1:** Studies contributing to the Research Topic.

**Authors**	**Research focus**	**Educational technology**	**Educational context**	**Analytic approach**	**Level of analysis**
Ebner and Gegenfurtner	Satisfaction	Webinar, asynchronous online course	Higher education, professional training	Meta-analysis	Individual
Francis et al.	Academic motivation, expectancy, value, cost, interest	Asynchronous online course	Higher education	Structural equation modeling	Individual
Järvenoja et al.	Socially shared regulation processes	Technology-supported learning environment	K12 education	Multimodal analysis of multichannel data	Group
Knigge et al.	Affective well-being and attitudes	Video-based online training	Teacher education	Latent change models	Individual
Martens et al.	Affective states, intrinsic motivation	Wearables	Higher education	Multimodal analysis of multichannel data	Individual
Molenaar et al.	Intentions for regulation	Adaptive learning technologies	K12 education	Analyses of variance	Individual
Näykki et al.	Emotional experiences	Social networks, games, digital fabrication	Early childhood, primary, higher education	Qualitative case analysis	Group
Pietarinen et al.	Affect	Virtual laboratory	K12 education	Video-based frequency analysis	Group
Quesada-Pallarès et al.	Motivational and self-regulation learning strategies	Asynchronous online course	Vocational education and training	Confirmatory factor analysis, multiple regression	Individual
Stephan et al.	Achievement emotions, technology acceptance	Asynchronous online course	Teacher education	Analyses of (co)variance	Individual
Testers et al.	Motivation to learn, intention to transfer	Asynchronous online course	Higher education	Structural equation modeling	Individual
Wortha et al.	Emotional profiles	Hypermedia-based tutoring system	Higher education	Person-centered and variable-centered cluster analysis	Individual

Reflecting on the future of research on affective learning in digital education, studies on the processes and products of affective learning in digital education will increasingly be based on multimethod and mixed method analyses of multimodal data, on individual and group levels of analysis. Digital technologies will continue to evolve fast, thus affording novel contexts both for educating learners and for collecting data of their (shared) affective learning processes. Major challenges lie in the synthesis of evidence on the effectiveness of technology-supported tools for digital and face-to-face education as well as in the integration of multimodal analyses of affective learning processes. This Research Topic addresses these challenges, which have gained further relevance during the COVID19 pandemic. We hope that you will enjoy reading the contributions as much as we did.

## Author Contributions

AG, SN, LF, SJ, and JH: writing, review, and editing. All authors contributed to the article and approved the submitted version.

## Conflict of Interest

The authors declare that the research was conducted in the absence of any commercial or financial relationships that could be construed as a potential conflict of interest.

